# 

**Published:** 1855-02

**Authors:** 


### RECEIPTS FROM JAN. 1 UP TO JAN. 27, 1855.

**ILLINOIS.**

| | |
|---|---|
| Dr John S Dewey | $5 00 |
| Z P Cabanis | 5 00 |
| W E Reynolds | 4 00 |
| W H Heller | 2 00 |
| M E Archer | 2 00 |
| M B Van Pett‹n | 2 00 |
| David T Kyner | 2 00 |
| Milton Bacon | 2 00 |
| J M Winn | 2 00 |
| Baily Ragon | 2 00 |
| C Goodbrake | 2 00 |
| F A Payne | 2 00 |

**WISCONSIN.**

| | |
|---|---|
| Dr James Cody | 2 00 |
| D F Shumway | 2 00 |
| D W Cailey | 2 00 |
| Dr H L Butterfield | $2 00 |
| S S Clark | 2 00 |

**IOWA.**

| | |
|---|---|
| Dr J D Blanchard | 5 00 |
| S L Hazen | 2 00 |
| William Watson | 2 00 |
| R W Hall | 2 00 |

**INDIANA.**

| | |
|---|---|
| Dr J A Adrain | 2 00 |
| James Ford | 2 00 |
| R A Cameron | 2 00 |
| E Jenison | 2 00 |

**MICHIGAN.**

| | |
|---|---|
| Mrs C A Dayton | 1 00 |

